# Editorial: Raising awareness around trends in noncommunicable diseases and their risk factors to promote global prevention and control

**DOI:** 10.3389/fpubh.2025.1553630

**Published:** 2025-01-28

**Authors:** Sonu Goel, Madhur Verma, Archisman Mohapatra, Stevo Popovic

**Affiliations:** ^1^Department of Community Medicine & School of Public Health, Post Graduate Institute of Medical Education and Research (PGIMER), Chandigarh, India; ^2^School of Medicine, Faculty of Education & Health Sciences, University of Limerick, Limerick, Ireland; ^3^Faculty of Human & Health Sciences, Swansea University, Swansea, United Kingdom; ^4^Department of Community and Family Medicine, All India Institute of Medical Sciences, Bathinda, Punjab, India; ^5^GRID Council, Noida, India; ^6^Faculty for Sport and Physical Education, University of Montenegro, Niksic, Montenegro; ^7^Balkan Institute of Science and Innovation, University of Montenegro, Podgorica, Montenegro; ^8^K-CLUB, Korea University, Seoul, Republic of Korea

**Keywords:** non-communicable diseases, risk factors, promotion, global prevention and control, raising awareness around trends

Non-communicable diseases (NCDs), encompassing cardiovascular diseases, cancer, diabetes, and chronic respiratory illnesses, have emerged as a significant global health challenge, exerting immense pressure on healthcare systems and economies worldwide. As per the World Health Organization (WHO), Non-communicable diseases (NCDs) globally account for 41 million deaths annually, resulting in 74% of global mortality (1). The NCD's contributions to the Disability-adjusted life years (DALY) are the highest globally and is the only disease group for which DALYs increased from 1.47 billion in 2010 to 1.73 billion years in 2021 (2). The multifaceted etiology of NCDs underscores the imperative for comprehensive approaches addressing underlying risk factors and quantifying their impact of exposure to key risk factors is necessary to inform public health policy and practice and help prioritize the use of scarce resources to reduce the existing disparities (3). According to WHO, NCDs arise from a combination of behavioral, metabolic, and environmental risk factors. Modifiable behaviors such as tobacco use, physical inactivity, unhealthy diets, and harmful alcohol consumption significantly elevate NCD risks (1). Metabolic risk factors, including high blood pressure, obesity, elevated blood glucose, and high blood lipids, drive NCD development. Environmental factors like air pollution further exacerbate NCD risks, causing 6.7 million deaths globally, with 5.7 million linked to conditions such as stroke, heart disease, chronic obstructive pulmonary disease, and lung cancer (1). While most of the risk factors are considered modifiable, efforts to minimize them are abysmally low worldwide. One crucial bottleneck to this sluggish response is a lack of awareness or insufficient knowledge about NCDs and their risk factors among people and policymakers.

Although these risk factors are considered modifiable, global efforts to minimize them remain abysmally low. A critical bottleneck in this sluggish response is the widespread lack of awareness or inadequate knowledge about non-communicable diseases (NCDs) and their associated risk factors among the general public and policymakers. This highlights the urgent need to prioritize raising awareness about NCDs and their risk factors.

Various articles in the special edition of Frontiers in Public Health highlight behavioral change interventions and associated barriers to mitigating NCDs. Zhou et al. have reported the direct effect of health literacy on a health-promoting lifestyle in their study. The article highlights the importance of educating people on increasing trends of NCDs without overwhelming that with information by Xu et al., who developed hypertension risk nomograms based on physical fitness parameters, advocated for personalized risk assessments, and promised to improve cardiovascular disease prevention by tailoring strategies to individual health profiles. ^ZnykandKaleta^ identified barriers that Polish general practitioners face in providing healthy lifestyle counseling, including lack of time, insufficient training, and patient resistance, highlighting systemic obstacles that need addressing to enhance preventive care.

Wang et al. presented a protocol for managing health risks in China that emphasizes regular health assessments, lifestyle modifications, and public awareness campaigns. Khan et al. presented the HEARTS technical package to control hypertension, which includes standardized treatment protocols and capacity-building initiatives, demonstrating a global commitment to managing cardiovascular risk factors. Leu et al. explored the impact of hard work and long hours on the health of Singaporean young adults, shedding light on how occupational stressors contribute to the growing burden of NCDs.

These studies collectively highlight the need for comprehensive strategies addressing lifestyle factors, systemic healthcare barriers, and educational interventions. In line with the existing studies in this Research Topic, the editorial summarizes the following initiatives to curb the rising burden of NCDs.

## Awareness campaigns and education

Education campaigns foster public understanding of NCDs and empower individuals to adopt healthier lifestyles. Leveraging diverse communication channels, including mass media, digital platforms, and community outreach initiatives, facilitates the dissemination of evidence-based information on NCD prevention and risk reduction strategies. Educational interventions targeting schools, workplaces, and local communities serve as catalysts for behavioral change and sustainable health practices (4). Improving the caliber and volume of counseling requires a health strategy that assists in removing obstacles and fostering an environment where general practitioners can successfully apply diet and physical activity counseling techniques.

## Policy advocacy and structural interventions

Effective NCD prevention necessitates a supportive policy environment conducive to health-promoting behaviors. Advocacy efforts aimed at context-specific policy formulation and its effective implementation play a pivotal role in curbing NCD prevalence and mitigating associated risk factors. Taxation policies on tobacco and sugar-sweetened beverages, food labeling regulations, and urban planning initiatives promoting active transportation and recreational spaces exemplify strategies for creating health-enhancing environments (5). The development of management skills across various healthcare workforce cadres, the availability of reasonably priced, high-quality, sustainable supply of antihypertensive medications, a robust digital health information system, and a robust primary healthcare infrastructure, including community-based services, are all necessary for the successful implementation of NCD prevention programs (6).

## Partnerships and collaborative initiatives

Collaborative endeavors involving governmental agencies, non-governmental organizations, academia, and the private sector are instrumental in advancing NCD prevention and control agendas. By harnessing collective expertise and resources, stakeholders can synergize efforts in raising awareness, implementing interventions, and advocating for policy reforms. Strategic alliances foster innovation, enhance programmatic efficiency, and optimize resource allocation toward sustainable health outcomes. A family-centric approach should be the mainstay of counseling services in order to improve ownership.

There is a need to reform the organizations' work culture. Extended periods of sitting, particularly in the workplace for young adults, can increase an individual's risk of developing hypertension and other NCDs. To help improve productivity, there should be a concept of Yoga or physical activity hour in the workplace (7). The idea of afternoon naps can be normalized in the workplace during lunch hours as they are known to prevent premature cognitive deterioration (8).

## Newer solutions

Addressing the complex interplay of NCD risk factors requires innovative solutions like artificial intelligence (AI). AI-based machine learning algorithms enable the analysis of vast amounts of health data to identify patterns, trends, and correlations related to NCDs and their risk factors. By mining electronic health records, epidemiological databases, and social media platforms, AI-powered analytics can uncover insights into disease prevalence, geographic disparities, and emerging health trends (9). These data-driven insights inform evidence-based strategies for raising awareness and tailoring interventions to specific population needs. Further, the Natural language processing (NLP) algorithms facilitate extracting and synthesizing relevant information from textual sources, including scientific literature, news articles, and online forums. Moreover, AI-driven chatbots and virtual assistants provide personalized health education, answering queries and dispelling misconceptions about NCDs in real-time. Predictive analytics models leverage AI algorithms to forecast future trends in NCD prevalence and identify individuals at high risk of developing these conditions. By integrating demographic, behavioral, and clinical data, predictive models enable early identification of predisposing factors and facilitate proactive interventions. Healthcare providers can leverage predictive analytics to tailor screening programs, pre-emptive interventions, and lifestyle counseling to individuals' unique risk profiles, optimizing resource allocation and improving health outcomes.

While AI offers promising NCD prevention and control opportunities, ethical considerations regarding data privacy, algorithmic bias, and equitable access must be carefully addressed. By harnessing AI-driven interventions, stakeholders can personalize interventions and empower individuals to make informed health decisions.

Addressing non-communicable diseases (NCDs) requires integrating traditional methods, such as public awareness campaigns and community programs, with innovative approaches like AI-driven tools. Traditional interventions are accessible and culturally relevant, while innovations enable personalized health recommendations and real-time monitoring. For example, school-based education can be amplified through digital platforms, and AI tools can monitor the impact of policies like tobacco taxation.

Collaboration among governments, NGOs, and private sectors is essential to bridge gaps in technology access and foster trust. This integrated approach ensures comprehensive, sustainable strategies to reduce the NCD burden while addressing health disparities.

To conclude, the world is uniformly going through a rapid demographic and economic transition at an overwhelmingly fast pace. Accounting for 74% of global deaths, NCDs pose a severe threat to health systems and economies. Thus urgent and timely actions are warranted to prevent the emergence of new cases and adequately manage the known cases. Insufficient knowledge regarding NCDs, their risk factors, and indifferent attitudes and practices hinder prevention efforts. This editorial proposed strategies to boost educational campaigns, undertake policy advocacy, and promote structural reforms to improve healthful behaviors. Partnerships among governments, NGOs, academia, and the private sector are crucial. Additionally, innovative solutions like AI can offer valuable insights and personalized interventions. A comprehensive, multifaceted approach is essential to reduce NCDs, address health disparities, and foster sustainable health outcomes globally.
